# Public Health and Prisons: Priorities in the Age of Mass Incarceration

**DOI:** 10.1146/annurev-publhealth-071521-034016

**Published:** 2022-12-21

**Authors:** David H. Cloud, Ilana R. Garcia-Grossman, Andrea Armstrong, Brie Williams

**Affiliations:** 1 Center for Vulnerable Populations, San Francisco School of Medicine, University of California, San Francisco, California, USA; 2 Rollins School of Public Health, Department of Behavioral, Social, and Health Education Sciences, Emory University, Atlanta, Georgia, USA; 3 College of Law, Loyola University New Orleans, New Orleans, Louisiana, USA

**Keywords:** prisons, mass incarceration, correctional health, decarceration

## Abstract

Mass incarceration is a sociostructural driver of profound health inequalities in the United States. The political and economic forces underpinning mass incarceration are deeply rooted in centuries of the enslavement of people of African descent and the genocide and displacement of Indigenous people and is inextricably connected to labor exploitation, racial discrimination, the criminalization of immigration, and behavioral health problems such as mental illness and substance use disorders. This article focuses on major public health crises and advances in state and federal prisons and discusses a range of practical strategies for health scholars, practitioners, and activists to promote the health and dignity of incarcerated people. It begins by summarizing the historical and sociostructural factors that have led to mass incarceration in the United States. It then describes the ways in which prison conditions create or worsen chronic, communicable, and behavioral health conditions, while highlighting priority areas for public health research and intervention to improve the health of incarcerated people, including decarceral solutions that can profoundly minimize—and perhaps one day help abolish—the use of prisons.

## INTRODUCTION

Mass incarceration is a pressing social problem with important implications for the fields of public health and medicine. For decades, the United States has incarcerated more of its population than have any other nations.^1^ The United States comprises less than 5% of the global population but, with nearly 2 million people behind bars, incarcerates 20% of all prisoners, and its 2021 incarceration rate (664 per 100,000 people) substantially exceeds rates in both democratic and authoritarian nations alike (68, 122). An extensive and growing body of research links mass incarceration to inequitable distributions of disease, despair, and death along lines of race and ethnicity, class, gender, and geography (154, 160). It shows that mass incarceration adversely impacts the health and well-being of people who directly experience it (66, 140) and compromises the health of families and communities, especially among Black, Indigenous, and People of Color (BIPOC) (97, 106, 158–160). Recent studies connect exposure to incarceration with a wide range of adverse health outcomes across socioecological levels (106, 139, 160). These include increased vulnerabilities to and rates of HIV (136), hepatitis C (136), sexually transmitted infections (107), opioid use disorder (83), drug overdose (100), age-related frailty (63), suicide (25), homicide (25), asthma (150), cardiovascular disease and hypertension (152), psychiatric morbidity (72), and COVID-19 (89, 93).

It is beyond the scope of this article to address the full spectrum of health-related issues across the vast US carceral enterprise (from police departments to immigration detention and probation/parole supervision). Instead, this article focuses on major public health crises and advances in state and federal prisons and discusses a range of practical strategies for health scholars, practitioners, and activists to promote the health and dignity of incarcerated people.^2^ We first summarize the public health–relevant historical and sociostructural factors that have led to mass incarceration in the United States. We then describe the ways in which prison conditions can create or worsen chronic, communicable, and behavioral health conditions, while highlighting priority areas for public health research and intervention to improve the health of incarcerated people, including decarceral solutions that can profoundly minimize—and perhaps one day eradicate—the use of prisons. The public health challenges and opportunities discussed in this article are informed by a thorough, yet nonexhaustive, review of extant literature as well as by our experiences as academic physicians, lawyers, and social scientists devoted to promoting the health and human rights of incarcerated people while fighting in parallel to dismantle carceral structures that perpetuate harm, violence, and health inequities.

## RECKONING WITH THE ROOTS OF MASS INCARCERATION

Understanding the historical antecedents and structural components of American mass incarceration is critical for developing interventions that are jointly focused on the acute symptoms and root causes of this public health crisis. In 2014, the National Academies of Science, Engineering, and Medicine published a report describing the legal, economic, and political forces that fueled the steep surge in American prison populations that began in the 1970s, peaked in 2009, and (despite some declines) has remained relatively stagnant on a national scale (144). The causal factors are complex but share common denominators: They are deeply rooted in centuries of the enslavement of people of African descent and the genocide and displacement of Indigenous people and are inextricably connected to labor exploitation, racial discrimination, the criminalization of immigration, and inadequacies in systems of care and support for behavioral health problems such as mental illness and substance use disorders (16, 46). In this setting, mass incarceration emerged not as a rational response to crime, but as a recalibration of legalized racism—a direct backlash to the social, economic, and political progress of the civil rights movement of the 1960s (73, 142). With a disproportionately adverse impact on BIPOC communities, mass incarceration has come to be known as the “new Jim Crow” (7). Understanding the roots of mass incarceration requires an understanding of the public health actions that inadvertently contributed to this era.

Some policies outside the criminal legal system have generated and reinforced America’s extreme reliance on police, courts, jails, and prisons for social and public health problems (90). In the 1960s, the medical and public health communities amplified calls for the deinstitutionalization of people with mental illness in favor of enhanced community-based care. This movement was further facilitated by advances in psychotropic medications, enhanced constitutional protections against civil commitment, the growing influence of community psychiatry, and the enactment of Medicaid (56). The Community Mental Health Act of 1963, which expanded mental health services through federal financing, intended to incentivize local communities to create alternatives to state asylums. Deinstitutionalization succeeded in downsizing the asylum population: Between 1955 and 2000, the total number of people held in state psychiatric hospitals decreased by 90% (60). However, creation of a vast network of community mental health centers fell short as federal policy makers reversed promises made in the Community Mental Health Act (56). The nation’s carceral footprint expanded concomitantly with decimations to community mental health and social welfare systems, helping to set the stage for jails and prisons to devolve into de facto institutions for people with chronic mental illness and substance use disorders (70).

Historians have traced how promising components of civil rights era federal programs, enacted to break cycles of intergenerational poverty and social inequalities in historically disenfranchised communities, were stymied and subsumed by a sociopolitical shift in the 1970s that transformed a bureaucratic infrastructure created for a “War on Poverty” into one waging a “War on Crime” and then a “War on Drugs” (73). The war on drugs broadly refers to laws and policies that criminalized drug use, militarized police departments, and resulted in the adoption of draconian laws that assigned lengthy prison terms for drug-related offenses (which particularly affected BIPOC communities), catalyzing mass incarceration (7, 74). The persistent drug war became an engine of mass incarceration waged by politicians who weaponized racialized rhetoric to rationalize an unpreceded expansion of carceral power, disproportionately within communities of color, at the expense of public investments in systems that could address social determinants of health.

By the 1980s and 1990s, the growth of the “prison-industrial complex” (45), a term used to describe the prison systems’ links to racial capitalism, metastasized in lockstep with deep cuts to funding for public education, supportive housing, early education, food assistance, and community mental health resources (57, 142, 143). Meanwhile, judicial decisions and legislation stripped people with criminal legal involvement from access to vital public services and the right to vote (85). Lawmakers steered billions of dollars to the building of new prisons, supporting rural economies where many prisons were built (57, 60). American prisons took a decisively punitive turn, divesting from prison-based educational, vocational, and rehabilitative programming (144). Correctional workforces became increasingly militarized and trained in tactics to surveil and mete punishment, rather than understanding or addressing the social, economic, and health-related needs of people in their custody (126).

The US incarceration rate was already higher than those of other wealthy democracies before its prison boom began in the early 1980s, and the gap between the United States and European rates grew significantly over the next several decades. As the United States elevated retribution and deterrence as its guiding principles in correctional practice, other nations (most notably in Scandinavia and Northern Europe) embraced a fundamentally different philosophical, legal, and organizational approach (145). For example, in the late 1980s and 1990s, Norwegian prisons faced mounting challenges, including worsening conditions, a culture of distrust and antagonism between staff and incarcerated people, violence, and high levels of recidivism. In stark contrast to the United States, the Norwegian government moved to overhaul the principles, values, and functionality of its entire correctional system to center rehabilitation over punishment. Scandinavian penal philosophy established that it is the legal duty of courts, not prisons, to impose punishments. The purpose of prison, it was determined, should be laser-focused on helping people to “become better neighbors” (145).

To be sure, a prison is still a prison, whether the prison is located in Norway or in the United States. There are well-founded criticisms of the Scandinavian penal system, including, among others, isolated and restrictive living conditions for people detained pretrial and heavy reliance on incarceration for certain convictions (e.g., drug-related crimes). But certain principles guide the Scandinavian prison systems to mitigate the worst abuses and harms observed for people incarcerated in the United States. For one, the Scandinavian systems adopted the humanistic principle of “normality,” that punishment is the deprivation of liberty only and that prison should resemble life outside as much as possible, thereby easing a person’s return to the community (133). Accountability and rehabilitation are primary goals, and incarcerated people are guaranteed all other rights (e.g., education, health care, and voting) to minimize the stigma of a legal record and maximize their potential for successful reintegration into the community.

As the Scandinavian model was developed, US prisons became increasingly overcrowded, dehumanizing, and violent environments with little to no external oversight or accountability for how they affected the lives or health of incarcerated people and their families or even the health and safety of communities (148). Indeed, mass incarceration in the United States has driven distinctly detrimental impacts on population health. For example, Wildeman (158) examined the effects of incarceration rates on population health across 21 wealthy democracies between 1981 and 2005 and found a significantly negative association between incarceration and life expectancy only in the United States.

## PUBLIC HEALTH’S AWAKENING TO INCARCERATION AS A DRIVER OF HEALTH INEQUITIES

Since 1980, the US Department of Health and Human Services (DHHS) has published a decennial report for its Healthy People Initiative, a national blueprint of policy priorities for improving population health, describing determinants of health, devising cross-sector strategies, and establishing metrics for evaluating public health (80). Finally, in 2010, one year after the US prison population reached its peak, the DHHS included incarceration as a determinant of health disparities and identified it as a priority policy area for reform (34). Since then, public health experts have increasingly called for bolder, structural, and policy-level changes to address the health impacts of mass incarceration (38, 40, 102). Such calls-to-action have been amplified by social movements focused on ending police violence, greater attention to the public health crisis that unfolded in jails and prisons during the COVID-19 pandemic, and a proliferation of research in public health and medicine unveiling linkages between incarceration and health inequalities. In the following sections, we highlight knowledge generated by the fields of public health and medicine about the public health challenges and harms of prison as well as the public health solutions that are being tested to address the American epidemic of mass incarceration.

## IMPORTANT AREAS OF FOCUS FOR PUBLIC HEALTH RESEARCH AND INTERVENTIONS

### Serious Mental Illness

Correctional facilities are the largest providers of publicly funded psychiatric services for people with serious mental illnesses (SMI) (70, 111). The prevalence of SMI is at least two to four times higher in state prisons than in the community (111). Yet, prisons generally lack sufficient clinicians and therapeutic environments to meet the needs of this population. People with SMI are especially vulnerable to exploitation and victimization by other incarcerated people and to mistreatment by prison staff. Correctional officers generally lack the training needed to recognize mental health symptoms and often perceive behaviors stemming from psychiatric conditions as willful violations of prison rules, resulting in punishment and, sometimes, violence. People with SMI are also more likely to experience placement in long-term solitary confinement, where they are highly vulnerable to its harms. Moreover, mental health treatment is often delivered at the cell front, through the slats of a cell’s door or bars, in earshot of other residents and staff, undermining confidentiality and the therapeutic relationship (5).

Addressing the overrepresentation of people with SMI in prisons will require substantial investments to expand community-based psychiatric care, housing, and social services to prevent arrests and incarceration and to facilitate continuity in care and support for people returning to the community from prison. The Affordable Care Act has increased behavioral health services in underserved communities in states that opted to expand Medicaid (155). Medicaid expansion has also contributed to reductions in police arrests (129). However, as of May 2022, 12 states still have not expanded Medicaid, a missed opportunity to shrink carceral contacts by enhancing access to community health care (48, 75). Indeed, states yet to expand Medicaid also tend to imprison the greatest percentages of their residents (163). Unlike the United States, nations in Europe and other democracies with nationalized health care systems benefit from better integration of community and correctional medicine. This approach helps people with SMI to access services that may protect them against involvement with the criminal legal system and also helps ensure that clinicians working in correctional settings have access to medical records to inform treatment regimens during and following periods of imprisonment.

US state health authorities have opportunities to improve the accessibility and quality of prison-based health care services by embracing Norway’s import model, which delegates the oversight and delivery of correctional health care to community-based providers (rather than departments of correction), thereby enhancing continuity in care, ensuring compliance with community standards, and incorporating incarcerated people into population health at federal, state, and local levels (4, 121). The import model may also be a viable strategy for addressing staffing shortages and high-turnover rates among clinicians in correctional settings by creating structures for community-based providers to deliver care to incarcerated people without working directly for departments of correction. Establishing and maintaining organizational divisions between clinical and correctional professionals in these settings may also help empower clinicians to adhere to ethical values and standards of public health practice and buffer against susceptibilities to “dual loyalties,” or the ethical dilemmas that health care professionals encounter when practicing in the prison setting that can result in medical practices that run afoul of ethical principles of medicine and public health (109).

### Suicide

Suicide is a leading cause of mortality in US prisons; between 2001 and 2018, prison suicides increased by 85% (34). These rates eclipse those in the community, which is likely a reflection of the high prevalence of adverse childhood experiences, economic instabilities, and mental health and substance use disorders among incarcerated people (54). Evidence suggests that incidents of nonsuicidal self-harm (e.g., cutting, swallowing objects, banging one’s head) occur nearly daily in US prison systems. Between 5% and 18% of men and 5% and 24% of women engage in self-harm during imprisonment (11). A range of clinical (depression, psychotic disorders, substance use), psychosocial (trauma, family dysfunction, histories of self-harm), and institutional-level factors have been linked to suicides in prisons, reinforcing important targets for public health interventions (54, 164).

Prisons are poorly equipped to prevent or respond to suicide and self-harm, and their policies and practices often precipitate vulnerabilities to self-injury and suicidality. Self-harm and suicide have been connected to institutional policies that intensify social isolation (e.g., placement in solitary confinement and absence of family visits) as well as overcrowding, victimization, and lack of staff supervision (17, 164). Some states define self-harm among imprisoned persons as a criminal offense or rule violation, meeting this health emergency with punishment rather than health care and compassion. For example, Louisiana law criminalizes “self-mutilation” with a penalty of up to two additional years in prison (LA Rev. Stat. § 14:404). Such laws and policies are prime for analysis and debunking by public health experts.

Together, such evidence underscores the importance of educating and training staff on suicide prevention and on how to respond with trauma-informed compassion in addition to emergency clinical services. Increasing access to mental health care and setting restrictions for solitary confinement and other punitive responses to self-harm and suicide attempts are also vital (5, 39). Adoption of a root cause analysis or sentinel events approach to incidents of self-harm would also enable prison systems to better understand organizational, contextual, and individual contributors to self-harm and suicide events and to develop multidisciplinary prevention and mitigation protocols (110).

### Substance Use Disorder and Overdose

People who use drugs (PWUD) are gravely overrepresented in US prisons; approximately 58% of people in state prisons have a substance use disorder. Drug markets operate within prisons, where people continue or begin using drugs for similar reasons as they would in the community (33). Overdose is a leading cause of death among currently and formerly incarcerated people. The number of people who died from “drug or alcohol intoxication” in prison increased over 600% from 2001 to 2018 (34), while people are 12.7 times more likely to die from an overdose during the first two weeks following prison release (23).

Most prisons do not employ community strategies to reduce fatal overdoses. Studies have shown a range of postrelease benefits, such as enhanced engagement in treatment, decreased drug use, reduced risk of fatal overdose, and lower recidivism, associated with providing medications for opioid use disorder (MOUD) (28, 58, 86, 127). However, access to MOUD (methadone, buprenorphine, naltrexone) during imprisonment and upon release remains inadequate (87, 141). Among 21 states with the heaviest burden of opioid-related mortality, buprenorphine and methadone were provided in only 15% and 9% of state prisons, respectively, and often only to subsets of patients (e.g., pregnant women) (123). Similarly, implementing overdose education and naloxone distribution (OEND) programs in prisons is an underutilized strategy for reducing overdoses (120). While some correctional staff carry naloxone and some facilities distribute it upon reentry (165), few state prison systems have OEND programs that are focused on reducing in-prison overdoses, and naloxone is rarely accessible to incarcerated PWUD, a missed opportunity to save lives (27, 138).

In prison, people suspected of, accused of, or found to be using drugs may be subjected to strip searches and forced (and sometimes unmonitored) detoxification, rather than receiving treatment and harm reduction strategies (44). In some cases, these individuals face new criminal charges that extend incarceration and/or disciplinary sanctions such as the loss of visitors or solitary confinement (132). PWUD in prison also lack access to harm reduction strategies for overdose (e.g., fentanyl testing strips, naloxone) and likely have less knowledge about the content of prison-market drugs. Such circumstances may lead to riskier drug use and disincentivize calling for help in an overdose. Moreover, punishments that diminish social supports may also increase the risk of overdose because exposure to solitary confinement has been linked to an increased risk of postrelease overdose (29). More studies are needed to contextualize how drug policies, enforcement, and prison conditions shape vulnerabilities to overdose during incarceration and postrelease (132, 149).

The United States is far behind many other countries when it comes to incorporating evidence-based practices in harm reduction and addiction medicine into its public health infrastructure. Fundamental changes in US drug policy are needed to reduce mass incarceration while mitigating drug-related harms. Proposed public health solutions have included legalizing or decriminalizing drug use (64), exploring safe-supply proposals to disrupt the ubiquity of fentanyl and other synthetic opioids in the drug supply (78, 99), expanding capacity for delivering evidence-based drug treatment and harm reduction services in prisons, and advocating for racial equity in drug policy contexts.

More robust reforms are necessary that focus on dismantling carceral structures created under the guise of the war on drugs and replacing them with community-driven solutions rooted in the science of public health and the principles of human rights, harm reduction, and racial justice. As a starting point, state and local public health officials might consider drawing lessons from nations such as Portugal, who declared the war on drugs a failure and enacted sweeping reforms that decriminalized the use, purchase, and possession of all illegal drugs that did not exceed an amount necessary for 10 days of use and created a public health agency focused on facilitating the engagement of PWUD in treatment (116). The US state of Oregon has adopted perhaps the most substantial reforms in the spirit of decriminalizing the use of all drugs and bolstering public health responses (105). However, despite evidence of some success (76), even Portugal’s model has faced scrutiny related to a continued reliance on imprisonment for some drug-related offenses, overpathologizing of drug use behaviors, and a reticence to embrace a progressive paradigm that is grounded in human rights and responsive to the diverse spectrum of drug use behaviors in society. Such critiques signal the need for innovation in drug policy within the United States and globally (65, 92, 116).

In the context of prison environments, at a minimum, prisons should institute OEND, MOUD, and syringe service programs, which are already considered essential public health interventions in some correctional settings, including in the US state of Rhode Island and Switzerland (49). Rhode Island has successfully implemented many of these strategies, including a vending machine that distributes naloxone (77); guides are available for prisons seeking to implement OEND programs (9, 156). Public health departments, harm reduction organizations, and nonprofit drug treatment providers can play a bigger role in training staff about substance use, harm reduction, and behavioral treatments for substance use in addition to ensuring that incarcerated people are able to connect to services at reentry.

### Infectious Diseases

Prison residents bear a disproportionate burden of infectious disease due to structural risk factors, such as overcrowding and congregate living, and individual risk factors, such as exposure to blood and bodily fluids through substance use, sexual contact, or tattooing. Viral hepatitis is one of the most common infectious diseases in prisons. More than 1 in 10 prison residents has been diagnosed with hepatitis C, even though the prevalence in the general population is only 1 in 100 (94). Sexually transmitted infections (STIs) are almost two times more common in prison populations and HIV is three times more common, as compared with the general population (94). In fact, HIV incidence is highest among people who have experienced multiple incarcerations, suggesting that the transition back to the community may be critically important for HIV prevention (61). Although condom use reduces the risk of STIs, condoms are banned in most prisons.

Transmissible respiratory infections have long been a risk in prisons (135), where crowded conditions and inadequate ventilation can amplify transmission (18). The prevalence of tuberculosis (TB) is increased in prisons by a factor of 12 (94). Transmissions to corrections staff also make TB an occupational health concern (91). Since 2020, COVID-19 has caused widespread outbreaks in prisons across the United States among both residents and staff, leading to an occupational health crisis (124). The COVID-19 pandemic has exemplified how the design and environments of prisons facilitate outbreaks (89). As of May 2020, an estimated 600,000 people incarcerated in US prisons and more than 200,000 prison staff have been infected with COVID-19; more than 2,800 incarcerated people and more than 275 staff have died (43). Throughout the first year of the COVID-19 pandemic, the incidence of infections was 3.3 times higher in prisons and the incidence of death from COVID-19 was 2.5 times higher compared with the general public (93).

Preventing the spread of infectious diseases in prisons requires robust public health interventions, including screening, prevention, and treatment (50). Prisons can encourage infectious disease screening upon entry using opt-out testing strategies, which lead to higher testing rates, compared with risk-based testing or testing upon request (19). While the Centers for Disease Control and Prevention recommend opt-out testing for HIV, only 19% of prison systems utilized it in 2014 (134). Prisons should also offer evidence-based prevention measures for STIs and HIV, including condoms, syringe-service programs, and pre-exposure prophylaxis medications (103, 157). To reduce the risk of outbreaks of respiratory-borne illnesses, such as COVID-19, prisons should adopt prevention measures including widespread vaccine delivery (119), optimized ventilation, surveillance testing, and, most importantly, significant population reductions to safely accommodate social distancing as well as humane medical isolation and quarantine (18, 36, 89). Prevention of infectious disease in prisons also protects the surrounding communities, as staff may unknowingly carry diseases from prison environments to their families and communities.

### Noncommunicable Diseases

People who are incarcerated have higher rates of noncommunicable diseases such as asthma, hypertension, kidney disease, and cancer (24, 98, 162). Individual, community, and structural factors preincarceration contribute to these higher burdens of disease. Such risk factors include diet, activity, and substance use (individual); adverse childhood experiences and trauma exposures (interpersonal/community); and limited health care access, housing insecurity, food insecurity, and racism (structural) (114). In addition, incarceration produces its own risk factors that likely exacerbate health outcomes due to acute and chronic stress from dehumanizing conditions, exposure to trauma and violence, poor diet, and variable access to physical activity and quality health care (95, 114). For example, incarcerated people have elevated rates of hypertension (162), which can be exacerbated if they are held in solitary confinement (161). The long-term morbidity, mortality, and health care costs resulting from these disparities remain understudied, although preliminary evidence suggests that these chronic health conditions translate into increased mortality risk for people in custody (81). The field of public health has an important opportunity to investigate the causal pathways between conditions of confinement and adverse health outcomes.

In many European nations, correctional systems are better equipped to offer a range of nutritious, enjoyable, and ethnically diverse food options. In some Scandinavian prisons, for example, incarcerated people have regular access to onsite grocery stores that provide a full variety of meat, produce, and other items as found in a community market. Residents shop for their own groceries and prepare their own meals, and most have the option of dining in communal spaces with peers and/or staff or dining privately in their living quarters or outdoors. (Residents also stock and run these stores, developing employable skills.) Health promotion and nutritional scientists focusing on food deserts and other inequities in the US food supply should play a more vocal role in advocating for access to higher-quality and more substantial food options for incarcerated people, as well as in interrogating correctional policies that deprive people of their right to healthy food.

### Women’s Health

Although fewer women are incarcerated than men, the United States still has the world’s highest incarceration rate of women (82). Women in prison, like their male counterparts, experience elevated rates of chronic and infectious disease and mental health conditions (137). Moreover, 75–95% of incarcerated women have experienced physical or sexual trauma (166), and thus trauma-informed care must be foundational for this population. Northern Ireland, in response to a high prevalence of trauma and poor access to education among incarcerated women, has reimagined its prison for women as a “secure college” in which all residents are referred to as “students” and offered a program of study and self-improvement during their incarceration (79).

While the Bureau of Justice Statistics collects data on the health of incarcerated people, it does not collect data on reproductive or gynecologic health beyond pregnancy rates (166). Other sources have established that rates of cervical dysplasia (30) and cervical cancer (117) are disproportionately high among incarcerated women. More evidence is needed to understand the impact that public health interventions, including human papillomavirus vaccination, cervical cancer screening, health education, health system surveillance, and continuity of care, can play in reducing this disparity.

Perinatal care in prisons is quite variable, and the United States does not adhere to many international care standards for incarcerated pregnant women. The Federal Bureau of Prisons ended routine shackling of pregnant people in 2009; however, this practice persists in several states (24), even though it is considered a human rights violation internationally (59). Moreover, the United States is one of only a few countries that routinely separates mothers from newborns, even though mother–baby units lower recidivism rates and promote healthy mother–child bonds and parental education (104). Ensuring that evidence-based, high-quality, dignified perinatal care is accessible to incarcerated women is critical to the promotion of maternal, child, and community health.

### Transgender Health

Transgender and nonbinary (TNB) individuals are overrepresented in correctional facilities, but their health outcomes have been grossly understudied. Correctional settings can be particularly traumatic for TNB people, due to the lack of recognition of their gender identity, transphobia, and victimization (147). These risks lead some prisons to use solitary confinement as a safety strategy; in the National Transgender Discrimination Survey, 7% of respondents who had been incarcerated reported being held in isolation due to their gender identity alone (62). Given the harms associated with isolation (described below), solitary confinement should not be used involuntarily for safety reasons alone. TNB individuals also often face barriers to receiving gender-affirming health care within prisons. While gender-affirming interventions, including hair removal, hormone therapy, surgery, and voice training, are deemed medically necessary by the World Professional Association for Transgender Health (41), many people who desire gender-affirming hormones or surgery face access barriers in prison (125). California prisons lead the way in this realm; they have generated clinical guidelines and decision support tools to ensure that health care professionals are equipped to deliver gender-affirming health care (32).

### Aging and Serious Illness

Over the last three decades, older adults have become the fastest-growing age group in US prisons (131). The “graying” prison population is a direct result of long sentences from “tough-on-crime” policies and the “war on drugs” (35). Many incarcerated people develop geriatric conditions at an earlier age than do their nonincarcerated peers (in their 50s), including mobility impairment, urinary incontinence, hearing impairment, and difficulty performing activities of daily living such as bathing or dressing (63). Incarcerated older adults also have a higher burden of chronic medical conditions than do their age-matched community peers (63, 131). This finding translates into high correctional costs, as older adults incur up to nine times the cost of younger adults in prison (6, 20).

Many incarcerated older adults die in prison. Although many incarcerated patients navigate terminal illnesses, prison-based health care professionals often lack knowledge about advance care planning (51). Many incarcerated people have not completed an advance directive, including identifying a health care proxy and specifying their medical wishes (51). Prisons face barriers to providing community standard end-of-life care, as few have geriatricians or palliative care clinicians (113), let alone hospice services. Federal prisons and nearly every state prison system have compassionate release mechanisms for incarcerated people to die outside of prison (52), yet rates of approval are low due to narrow eligibility criteria, extended review processes, and the influence of nonmedical criteria, such as the nature of the crime or the individual’s prison disciplinary record (1, 101). The COVID-19 pandemic, which has disproportionately affected older adults, has renewed interest in improving compassionate release policies so that fewer people die behind bars (88).

Addressing the aging crisis in prisons requires a multifaceted approach. Correctional health care professionals should be trained to manage geriatric conditions and engage their patients in advance care planning, and health care professionals in community hospitals must have knowledge about how to provide ethical care to incarcerated patients (118). To achieve community standard care, all seriously ill patients should have access to palliative care and hospice services. Given increasing deaths in custody, increased mortality risk during the COVID-19 pandemic, soaring health care costs, and the low recidivism rates among the elderly (67), accessible and more expansive compassionate release programs are imperative. Public health researchers and advocates should call for bold sentencing reforms since long-term sentences do not improve public safety or reduce recidivism (15, 21), prison environments exacerbate morbidity, and second-chance mechanisms (including universal parole eligibility, presumptive parole, and second-look sentencing) can improve the rates of release among older adults and reduce burdens on prison health care systems (31).

### Solitary Confinement

Solitary confinement epitomizes the punitive and dehumanizing conditions that have come to define American prisons. Solitary confinement is the continuous exposure to social isolation and material and sensory deprivation for at least 22 h per day, often in a cell the size of a parking space. Access to clinical care, visits with family or friends, physical exercise, and other means of communication is minimal or nonexistent. Exposures to violence and chemical agents are common (39). At least 60,000–80,000 people (~4.5% of people incarcerated in US state prisons) are held in solitary confinement daily (22). People with SMI or cognitive or developmental disabilities and racial, ethnic, sexual, and gender minorities are all overrepresented in solitary confinement (115). Moreover, health care professionals working in solitary confinement units often experience dual loyalties: advocacy for one’s patient who is experiencing torture or allegiance to one’s employer (5). The general consensus in public health and medicine is that solitary confinement is a profoundly harmful practice, often leading to severe psychological distress, self-injury, suicide, impaired vision, and increased health-related costs, among other harms (69).

Public health research has helped advance litigation and legislation to reduce or end the use of solitary confinement. It has also led to the establishment of the United Nations Nelson Mandela Rules, which identify solitary confinement as torture under international law (146). Public health research should now shift from establishing the harms of this practice to assessing the impact of proposed solutions. For example, an assessment of reforms in North Dakota described policy changes that were inspired by Norwegian principles and yielded a 74.3% reduction in solitary confinement over 5 years accompanied by decreased violence and improved well-being for incarcerated people and staff alike (37). Ending solitary confinement will require creating clinically oriented interventions that transition people harmed by long-term solitary confinement into supportive environments, establishing proactive and trauma-informed approaches to responding to mental health crises, and developing alternative ways to hold people accountable who commit acts of violence. As more correctional systems become legally required to diminish their use of solitary confinement, public health professionals can play a pivotal role in creating alternative approaches to addressing violence and mental health crises that have been historically managed using solitary confinement.

While solitary confinement is a global human rights problem, Norway developed an intervention, the resource team, which is focused on enhancing services and resources for incarcerated persons who require physical separation for their own safety and the safety of others, mostly due to recent involvement in serious acts of violence and/or tendencies for self-isolation and vulnerability in congregate settings. This intervention, which began at Ila prison in Norway, is delivered by specially trained teams of clinicians and correctional officers who deliver recreational, educational, and vocational activities that are tailored to the needs of each person on a daily basis. The task of the resource team is to promote social interaction, establish trusting relationships, and minimize the occurrence of situations that may result in violence through enhanced de-escalation techniques. In collaboration with the Norwegian Correctional Service, the Amend program at the University of California School of Medicine is in the early stages of piloting resource teams in several prisons in the US states of Washington, Oregon, and California (https://amend.us/). Adapting and scaling such an intervention to the diverse needs and contexts of US prisons have potential for paving a path toward solitary confinement abolition by replacing its core components (isolation, idleness, and deprivation) with meaningful human interaction, compassion, and support. While more research is needed, preliminary and anecdotal evidence suggests that this approach leads to improvements in the health and well-being of incarcerated people and frontline correctional staff alike.

### Transitional Health Care

Most people confined to prison will eventually be released, including ~600,000 people each year. People released from prison face a range of social, economic, legal, and logistical barriers to stably reintegrating into society. Many struggle to find housing, employment, health care, and other essential resources, which increases vulnerabilities to premature death, worsening health status, and reincarceration (23, 96).

Collaboration with community health systems is critical for reducing the complex barriers to health care access experienced by people returning to their communities. Yet, many people return to society without health insurance, doctor’s appointments, medical records, or a continuity of care plan. The Transitions Clinic Network (TCN), a coordinated care model between correctional and community health care systems, has demonstrated that proactive linkages to primary care upon release leads to lower acute care utilization and decreased costs (71, 128). Wraparound programs that provide health care and assist with housing, education, and employment are promising interventions for people released from prison (35). Hiring formerly incarcerated community health workers to help people navigate health care and social services during reentry is an essential component of TCN that state public health systems can expand to reduce health inequalities and build bridges between community and correctional health providers (71, 128). State officials should revitalize or expand initiatives for ensuring that people are enrolled into insurance programs before release from prison and create capacity in state and local health systems that is tailored to the needs of currently and formerly incarcerated people and their community contexts.

### Lack of Data and Transparency

The near absence of external oversight bodies to monitor conditions in US prisons is another feature of American exceptionalism in its exertion of carceral power. Most other Western democracies have independent bodies that are responsible for inspecting and monitoring prison conditions to hold officials accountable for actions that threaten the health and safety of incarcerated people, in accordance with their adoption of the United Nations Optional Protocol for the Convention Against Torture. Although incarceration is part of the American fabric, research about the health and health care needs of incarcerated people is profoundly underdeveloped. First, just a tiny fraction of the National Institutes of Health (NIH) budget is allocated for projects designed to benefit incarcerated people, and few publicly accessible health data sets include information about people who are incarcerated (2, 3). The lived experiences of incarcerated people are largely excluded from the disease registries and Census-based data sets that research and government leaders rely on for monitoring disease, morbidity, and mortality (151). Federal agencies, such as the Bureau of Justice Statistics, rely on episodic voluntary surveys by correctional facilities for public health surveillance in prisons. However, data collection has been hampered by shifting agency responsibilities, extended publication delays, and changing data instruments. Imprisoned people are also omitted from federal statistics about unemployment, education, and economic insecurities (108). This exclusion from standard data collection efforts undermines efforts to understand the social and economic conditions that shape negative health outcomes associated with incarceration (108, 151). Thus, decisions about when, how, where, and to whom public health resources are allocated often fail to address the health of incarcerated people.

Second, despite overwhelming evidence of a public health crisis within prisons, laws and policies shield prisons from public view. Courts regularly defer to prison administrators in decision-making, including decisions denying family, community, and media contact with incarcerated people, and limit the ability of incarcerated people to advocate on their own behalf (13, 53). Comprehensive, independent oversight of US prisons is rare (47). Meanwhile, litigation, traditionally a tool used to expose conditions of incarceration, was limited in 1996 by the Prison Litigation Reform Act, resulting in a dramatic decrease in the number of prison-related lawsuits (53, 55). The lack of transparency around prisons is even more pronounced within correctional health care. Researchers must often navigate an array of legal and bureaucratic barriers to access prison health data. Most state and local governments also lack public health surveillance systems, internal performance metrics, and integrated data systems for monitoring the health of incarcerated people (2).

There is a dire need for legislative reforms that repeal existing barriers and create robust mechanisms for shedding light on the horrific conditions that persist behind prison walls, holding those in power accountable for mistreatment and abuse and facilitating public conversations about the urgency of decarceration and closures of prisons whose conditions and practices compromise the health and safety of the people they confine (12). Since 2010, a growing number of states have created new bodies or enhanced existing mechanisms for prison oversight after decades of advocacy. However, only 15 states plus the District of Columbia have created external mechanisms, which vary in their mission, structure, and power, for responding to complaints of incarcerated persons and/or for monitoring and reporting on conditions of confinement. New Jersey and Washington have statutory frameworks that may serve as a model for other states; they afford an ombudsman office the right of access to prisons, a duty to monitor and investigate complaints of incarcerated persons, and a mandate to produce public reports (47).

Public health leaders can contribute to efforts to enhance oversight of prison conditions in several ways. First, clinicians and epidemiologists can help advocate for legislation that seeks to establish oversight mechanisms by calling attention to its importance as a public health imperative and draws on lessons learned from the COVID-19 pandemic. Second, public health researchers can also contribute to monitoring duties of oversight bodies by developing reliable instruments for assessing the health implications of prison conditions and for monitoring the incidence of health conditions among incarcerated people. They can also leverage administrative data from correctional systems and merge them with community-health data sets to empirically assess relationships between prison conditions and morbidity and mortality among currently and formerly incarcerated people. Third, an emerging body of research is examining mass incarceration and prison conditions through a lens of environmental justice (14, 112). As threats of climate change mount, environmental scientists have an important role to play in assessing and addressing public health problems related to environmental hazards, such as extreme heat, polluted water, exposures to lead or other contaminants, and emergency responses to hurricanes, wildfires, and other natural disasters (26, 130). The theories, methods, and ethics of epidemiology, as the core science of public health, are vital tools for exposing health inequities that evade the nation’s public health surveillance systems.

## DECARCERATION AND ABOLITION AS PUBLIC HEALTH PRIORITIES

The field of public health is increasingly calling for structural-level changes to address the health impacts of mass incarceration. This trend is explicit in policy statements of professional organizations calling for an array of solutions, ranging from reforms to large-scale decarceration, and to the abolition of police, jails, and prisons. The civil unrest and political discourse following the murder of George Floyd created more public awareness of the daily injustices inflicted by the criminal legal system under the mirage of public safety. For instance, in response to police violence against Black communities, the American Medical Association “recognize[d] police brutality as a manifestation of structural racism disproportionately impacting Black, Indigenous, and other people of color” and directed physicians “to take steps to tackle policing reform and racial injustices” (8). Similarly, the American Public Health Association adopted a policy statement that articulated steps for “moving toward the abolition of carceral systems and building in their stead just and equitable structures that advance the public’s health” (10).

Addressing the issues raised in this article will require collective engagement from policy makers, practitioners, and activists working across public health’s subdisciplines. Decarceration, or depopulation, recognizes that both individual and community health would be improved if prisons contained fewer people. Decarceration calls for interventions that slow admissions to jails and prisons, reevaluates which crimes require incarceration over other solutions, shortens sentence terms, and accelerates releases. The COVID-19 pandemic inflicted disparate degrees of illness and death within crowded and hazardous conditions perfectly suited for widespread contagion, which forced health officials to recognize and policy makers to act on decarceration as an anticontagion imperative (153). Some systems listened to public health experts and community activists who called for decarceration. For example, district attorneys, police chiefs, and corrections staff implemented changes to reduce the population of jails, including temporary moratoriums on low-level arrests, cash bail, and technical probation violations for drug-related and other minor criminalized behaviors. These reductions in jail populations led to subsequent reductions in prison populations as well (122).

Some prison systems also took steps to facilitate early releases. A handful of state governors utilized executive orders or commutation powers to release people nearing the end of their prison term. Corrections and parole agencies in some jurisdictions granted compassionate releases or medical furloughs, and, in response to lawsuits, some judges ordered courts to identify and/or release people meeting specified criteria based on the nature of convictions and susceptibilities to COVID-related morbidity and mortality. While these actions resulted in thousands of people spending less time in prison, they netted nominal reductions to the size of the carceral state’s footprint. Moreover, most of the decarceration strategies were temporary. In many jurisdictions, police and prosecutors have resumed jailing people in situations that were banned or limited during the pandemic; the assembly line of mass incarceration has resumed (122).

The fight for decarceration during the pandemic offers invaluable insights, however. For example, the panoply of policies focused on reducing arrests, prosecutions, and admissions on the front end of the criminal legal system accounted for a greater share of jail and prison population reductions than did those that accelerated early releases. From a public health perspective, this observation signals the critical importance of continuing to press for legal interventions designed to minimize or abolish the role of police, courts, and correctional systems in responding to social and public health problems (42, 50, 122, 153).

## THE PROMISE OF PUBLIC HEALTH IN ADDRESSING MASS INCARCERATION

Downsizing or eliminating carceral systems must be paired with an unwavering commitment to expanding community-level supports for people most likely to encounter the criminal legal system. Calls for decarceration must reckon with the racially and economically oppressive origins of laws that continue to criminalize poverty, homelessness, and drug use. Dismantling carceral structures will require tireless public health education and activism that fosters societal reckoning with the criminal legal system’s inextricable ties to racialized violence and economic oppression, as well as the public health field’s complicities in abdicating many of its foundational responsibilities to police, courts, jails, and prisons. Public health educators must incorporate mass incarceration into the core curricula of public health schools. Philanthropic and government funding is needed to expand the work of researchers and organizers who are helping address social problems in their neighborhoods without involvement of the criminal legal system and who are addressing the health crises unfolding inside US prison systems.

The theoretical frameworks, methodological tools, and ethical tenets of epidemiology, sociobehavioral sciences, environmental sciences, and biostatistics are invaluable assets for documenting the harms of carceral structures but, more importantly, for awakening others to the possibilities of alternative solutions to socioeconomic and environmental problems besides incarceration. It is time for the field of public health to organize its efforts to combat the intersectional threats to our collective public health that are posed by carceral logic and prisons. A more just and healthy society will not be realized without attention to the epidemic of mass incarceration. The health and longevity of our society cannot afford to wait.

## Footnotes

1 The incarceration rate in El Salvador surpassed that of the United States in 2022 following a government campaign that has resulted in mass arrests of citizens.

2 While prisons and jails both share many of the same challenges, jails primarily detain people serving short sentences. Prisons, by contrast, confine people serving sentences ranging from one year to life, including those sentenced to the death penalty (in 24 states). Jails are typically geographically proximal to the communities where the people in custody live and work or where they were arrested. Prisons are often farther away from urban centers, and while ~600,000 people are released annually, prison populations are more stagnant than jail populations. In some states with especially large prison populations, a substantial percentage of people sentenced to prison terms for greater than one year are housed in local jails, in part due to limited capacity within state-operated prisons. For example, more than half of people sentenced to prison in Louisiana are physically housed in parish jails.
